# Construction and validation of a nomogram for patients with pancreatic neuroendocrine tumors: A population study of 5,927 patients

**DOI:** 10.3389/fgstr.2022.1088133

**Published:** 2023-01-10

**Authors:** Gaobo Huang, Weilun Song, Yanchao Zhang, Bingyi Ren, Yi Lv, Kang Liu

**Affiliations:** ^1^ National Local Joint Engineering Research Center for Precision Surgery and Regenerative Medicine, First Affiliated Hospital of Xi’an Jiaotong University, Xi’an, Shaanxi, China; ^2^ Department of Oncology, Xi’an No.3 Hospital, Xi’an, Shaanxi, China; ^3^ Department of Hepatobiliary Surgery, First Affiliated Hospital of Xi’an Jiaotong University, Xi’an, Shaanxi, China

**Keywords:** nomogram, cancer-specific survival, pancreatic, neuroendocrine tumors, prognosis

## Abstract

**Background:**

Pancreatic neuroendocrine tumors (pNETs) are a group of uncommon tumors derived from peptide neurons and neuroendocrine cells, and account for roughly 2% to 4% of all pancreatic neoplasms. This study aimed to construct and validate a nomogram for predicting the prognosis of patients with pNETs. Our data came from the SEER database.

**Methods:**

A total of 5927 pNETs patients between 2004 and 2018 were included in this study. The nomogram was constructed base on eight prognostic factors and validated by C-index, ROC curve and calibration curves. A nomogram based on eight independent prognostic factors (patient age, sex, race, tumor grade, AJCC T, AJCC N, AJCC M, surgery, radiation, chemotherapy, tumor function and marital status) was developed for the prediction of CSS at 3 and 5 years.

**Results:**

The C-index and AUCs of the nomogram demonstrated that its superiority in discrimination over AJCC staging system. The calibration plots showed the good consistency between predictions and actual observations.

**Conclusions:**

In conclusion, our nomogram could better predict the prognosis of pNETs patients than AJCC staging system. The nomogram could be improved by integrating more important factors other than SEER database.

## Introduction

Pancreatic neuroendocrine tumors (pNETs) are a group of uncommon tumors derived from peptide neurons and neuroendocrine cells, and account for roughly 2% to 4% of all pancreatic neoplasms (1, 2). The incidence of pNETs has increased from 1.07 to 5.25 per 100,000 in the United States over the past years (3). While few functioning pNETs secret hormones such as insulin, gastrin, glucagon, or other hormones and cause symptoms, most pNETs are nonfunctional (4). Due to the relative low incidence and non-functionality of pNETs, the prognosis of pNETs patients was rarely studied. Therefore, the prognosis of patients with pNETs is necessary to be further studied to help doctors and patients better understand this uncommon disease.

Currently, the American Joint Committee on Cancer (AJCC) TNM staging system is the most commonly used classification system for patients with pNETs (5). However, many significant prognostic factors such as gender, age, and grade were not included in AJCC system (6). Nomogram is an easy-to-use predictive system which is based on statistical model (7). It could be used to predict the prognosis of a disease by considering every selected factors, therefore this tool has been widely applied in clinical use (8–10).

This study aimed to construct and validate a nomogram for predicting the prognosis of patients with pNETs. The incorporated prognosis factors were obtained from the Surveillance, Epidemiology, and End Results (SEER) database. And we further compared our nomogram with AJCC staging system to determine whether this model provides more accurate prediction.

## Materials and methods

### Ethics statement

Our data came from the SEER database and signed a data agreement (11187-Nov2021), so our study was exempt from ethical review. This article does not contain any studies with human participants performed by any of the authors.

### Study population

**Figure 1:**
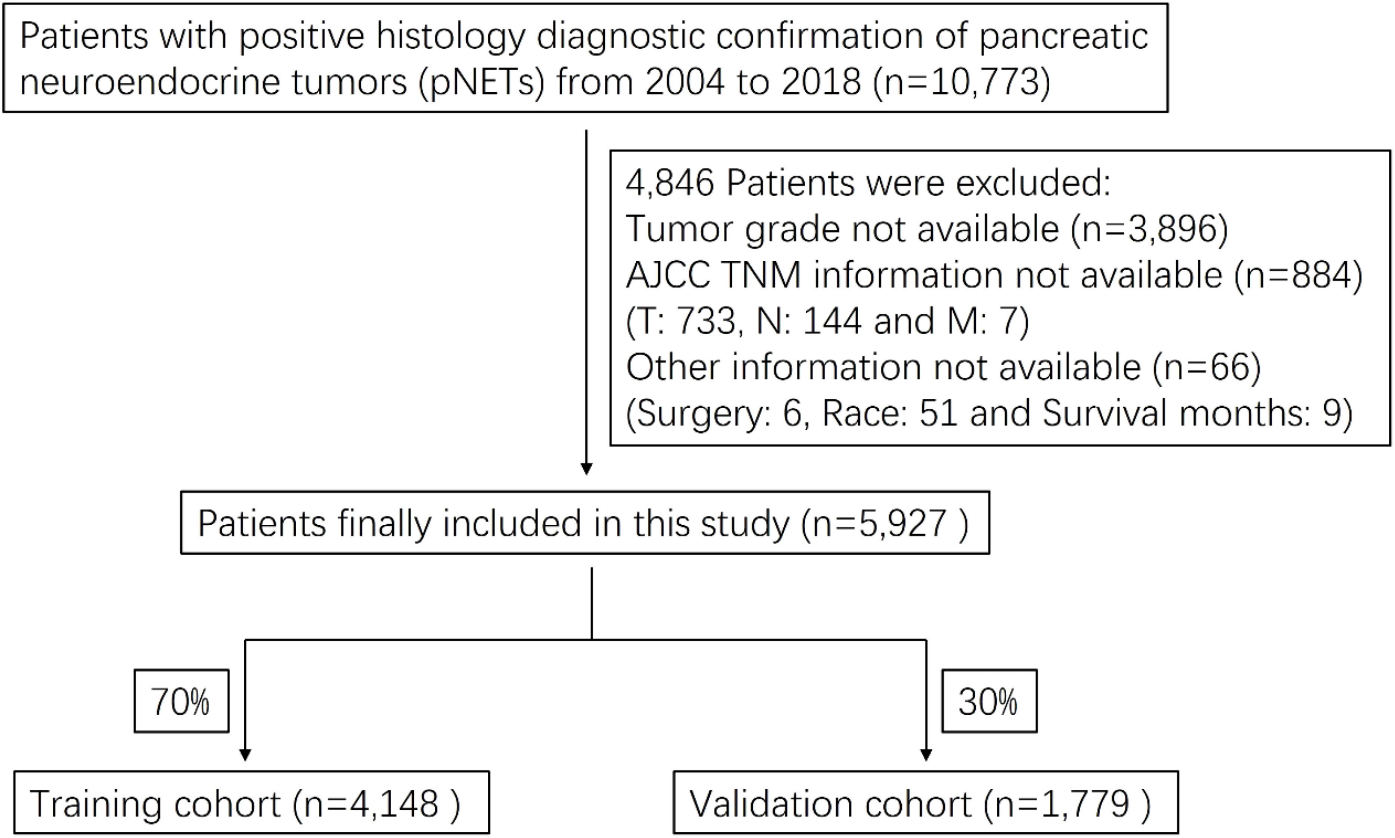
Flow chart of study patients’ enrollment.

We obtained patient data from the SEER Research Plus Data, 18 Registries, Nov 2020 Sub (2000-2018) incidence database, using SEER*Stat version 8.3.9. A total of 10,773 pNETs patients were identified and 5,927 patients were finally included in this study. The flow chart for selecting research samples was shown in **Figure 1**. The following variables were used in the analysis: patient age, sex and race; Tumor grade, AJCC staging for the extent of tumor (T), extent of spread to lymph nodes (N), and presence of metastasis (M); Surgery (Y/N), radiation (Y/N) and chemotherapy (Y/N); Tumor function and marital status.

The 3rd edition International Classification of Disease for Oncology (ICD-O-3) histology codes was used to select functioning and non-functioning pNETs. The functioning pNETs contained: Insulinoma (8151/3), Glucagonoma (8152/3), Gastrinoma (8153/3), Vipoma (8155/3), Somatostatinoma (8156/3), Enteroglucagonoma(8157/3) and ACTH-producing tumor (8158/3). NFpNETs contained: 8013/3, 8150/3, 8240/3-8246/3 and 8249/3.

### Statistical analysis

Endpoint for the current analysis was cancer-specific death (CSS). For nomogram construction and validation, we randomly divided all the pNETs patients into training (n = 4,148) and validation (n = 1,779) cohorts, in a ratio of 7:3 (11, 12). Multivariate Cox proportional hazards regression analysis was performed to identify variables (P < 0.05) that significantly affected CSS in the training group. Using these identified prognostic factors, we constructed a nomogram for predicting 3- and 5-year CSS rates in pNETs patients.

The nomogram was validated internally in the training cohort and externally in the validation cohort. We also developed a risk stratification system based on the total points of each patient in the training cohort. The cutoff value of the total points was calculated by X-tile software (version 3.6.1). To evaluate the discriminative ability of the nomogram, we used the concordance index (C-index) and the receiver operating characteristic (ROC) curve and assessed the area under the curve (AUC) (13, 14). A C-index or AUC of 0.5 indicates a discrimination ability that is no better than chance, whereas that of 1.0 indicates a perfect discrimination ability (15). Calibration curves were constructed using a bootstrap approach, with 500 resamples, to compare the predicted CSS with the CSS observed in the study.

All statistical analyses were conducted using SPSS (version 24.0; SPSS, Chicago, IL, USA) and R software (version 4.1.2; http://www.r-project.org/). A P value of less than 0.05 was considered to indicate statistical significance.

## Results

### Patient characteristics

A total of 5,927 pNETs patients from 2004 to 2018 were included in this study. The training and validation cohorts consisted of 4,148 and 1,779 cases, respectively, selected by the random split-sample method (split ratio: 7:3). In the total cohort of pNETs patients, the majority of patients were under 65 years old (59.0%), male (55.3%), and white (78.8%). Furthermore, most of the patients had well-differentiated grade (90.1%), T1 (34.1%), N0 (72.9%), and M0 (79.4%). Most of pNETs patients underwent surgery (75.4%), but only a small proportion of the patients received radiation therapy (3.36%) and chemotherapy (13.7%). 99.0% of pNETs were non-functional and a large number of pNETs patients were married (63.2%). The characteristics of pNETs patients in the training and validation cohorts were similar to those in the total cohort (**Table 1**).

**Table 1:** pNETs Patient characteristics in the study.

Characteristics	Total cohort	Training cohort	Validation cohort
	5927 (100%)	4148 (70%)	1779 (30%)
Age			
<65	3496 (59.0%)	2461 (59.3%)	1035 (58.2%)
≥65	2431 (41.0%)	1687 (40.7%)	744 (41.8%)
Sex			
Male	3280 (55.3%)	2284 (55.1%)	996 (56.0%)
Female	2647 (44.7%)	1864 (44.9%)	783 (44.0%)
Race			
W	4668 (78.8%)	3270 (78.8%)	1398 (78.6%)
B	673 (11.4%)	469 (11.3%)	204 (11.5%)
AI	34 (0.57%)	21 (0.51%)	13 (0.73%)
API	552 (9.31%)	388 (9.35%)	164 (9.22%)
Grade			
low	5341 (90.1%)	3739 (90.1%)	1602 (90.1%)
high	586 (9.89%)	409 (9.86%)	177 (9.95%)
AJCC T			
T1	2019 (34.1%)	1405 (33.9%)	614 (34.5%)
T2	1969 (33.2%)	1371 (33.1%)	598 (33.6%)
T3	1597 (26.9%)	1126 (27.1%)	471 (26.5%)
T4	342 (5.77%)	246 (5.93%)	96 (5.40%)
AJCC N			
N0	4322 (72.9%)	3018 (72.8%)	1304 (73.3%)
N1	1605 (27.1%)	1130 (27.2%)	475 (26.7%)
AJCC M			
M0	4706 (79.4%)	3282 (79.1%)	1424 (80.0%)
M1	1221 (20.6%)	866 (20.9%)	355 (20.0%)
Surgery			
No	1458 (24.6%)	1019 (24.6%)	439 (24.7%)
Yes	4469 (75.4%)	3129 (75.4%)	1340 (75.3%)
Radiation			
No	5728 (96.6%)	4004 (96.5%)	1724 (96.9%)
Yes	199 (3.36%)	144 (3.47%)	55 (3.09%)
Chemotherapy			
No	5117 (86.3%)	3570 (86.1%)	1547 (87.0%)
Yes	810 (13.7%)	578 (13.9%)	232 (13.0%)
Tumor function			
non-functioning	5865 (99.0%)	4104 (98.9%)	1761 (99.0%)
functioning	62 (1.05%)	44 (1.06%)	18 (1.01%)
Marital status			
Married	3745 (63.2%)	2633 (63.5%)	1112 (62.5%)
Single	2182 (36.8%)	1515 (36.5%)	667 (37.5%)

W, White; B, Black; AI, American Indian/Alaska Native; API, Asian or Pacific Islander; Low grade: grade I and II, high grade: grade III.

### Screening for prognostic factors of CSS

**Figure 2:**
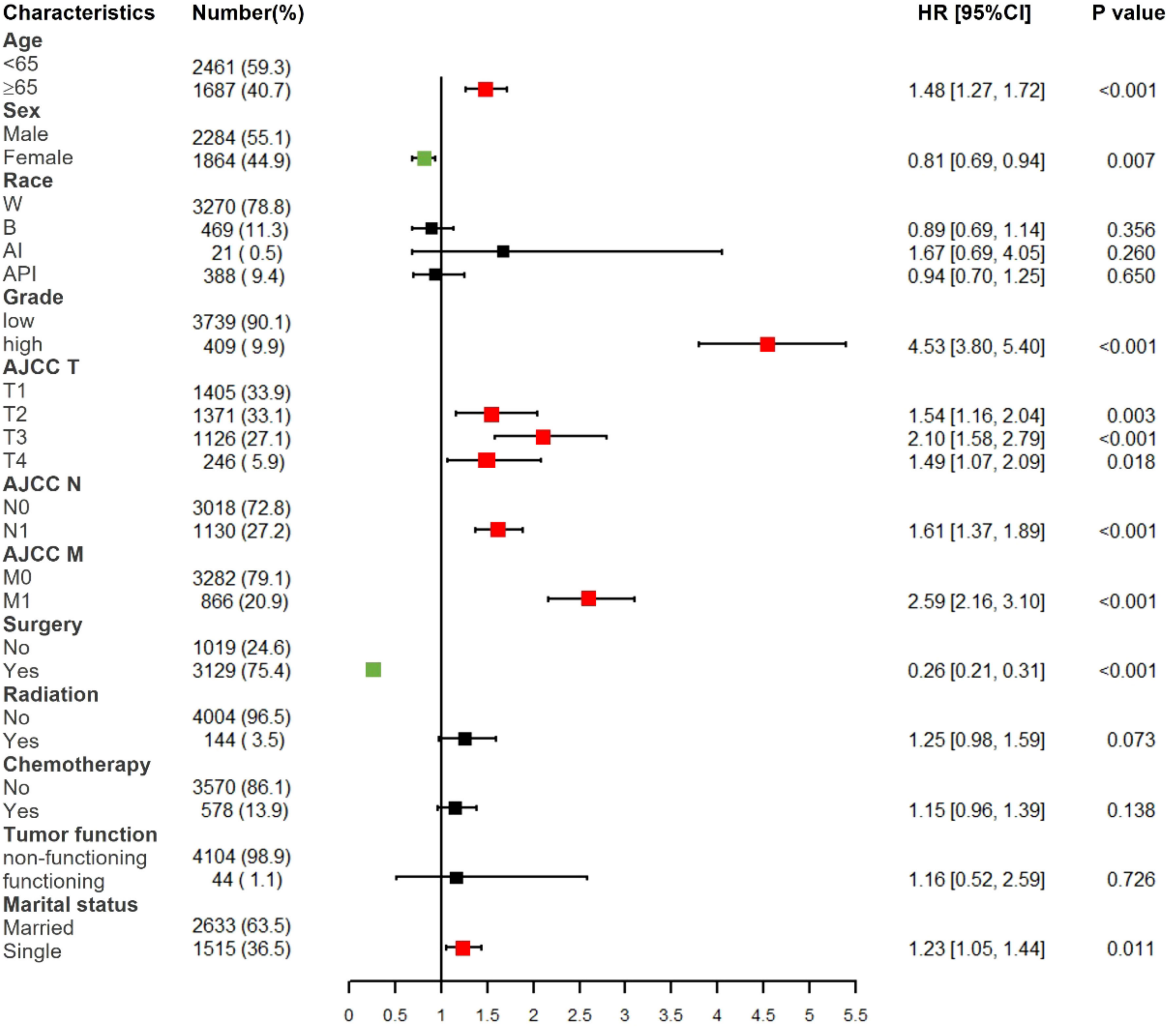
Forest plot of multivariate Cox regression analysis for CSS in pNETs patients. W, White; B, Black; AI, American Indian/Alaska Native; API, Asian or Pacific Islander; Low grade: grade I and II, high grade: grade III.

Based on multivariate Cox proportional hazards regression analyses, we identified eight independent prognostic factors in the training cohort. Over 65 years old (hazard ratio [HR] = 1.48, P < 0.001), female (HR = 0.81, P < 0.01), poorly differentiated grade (HR = 4.53, P < 0.001), AJCC T2/T3/T4 (HR = 1.54/2.10/1.49, P < 0.01), AJCC N1 (HR = 1.61, P < 0.001), AJCC M1 (HR = 2.59, P < 0.001), surgery (HR = 0.26, P < 0.001) and single status (HR = 1.23, P < 0.05) were all significantly associated with CSS in pNETs patients (**Figure 2** and **Table 2**).

**Table 2:** Multivariate Cox regression analysis based on all variables for pNETs patient cancer-specific survival (Training Cohort).

Characteristics	Multivariate analysis	
	HR [95% CI]	P value
Age		
<65	Reference	
≥65	1.48 [1.27, 1.72]	<0.001***
Sex		
Male	Reference	
Female	0.81 [0.69, 0.94]	0.007**
Race		
W	Reference	
B	0.89 [0.69, 1.14]	0.356
AI	1.67 [0.69, 4.05]	0.260
API	0.94 [0.70, 1.25]	0.650
Grade		
low	Reference	
high	4.53 [3.80, 5.40]	<0.001***
AJCC T		
T1	Reference	
T2	1.54 [1.16, 2.04]	0.003**
T3	2.10 [1.58, 2.79]	<0.001***
T4	1.49 [1.07, 2.09]	0.018*
AJCC N		
N0	Reference	
N1	1.61 [1.37, 1.89]	<0.001***
AJCC M		
M0	Reference	
M1	2.59 [2.16, 3.10]	<0.001***
Surgery		
No	Reference	
Yes	0.26 [0.21, 0.31]	<0.001***
Radiation		
No	Reference	
Yes	1.25 [0.98, 1.59]	0.073
Chemotherapy		
No	Reference	
Yes	1.15 [0.96, 1.39]	0.138
Tumor function		
non-functioning	Reference	
functioning	1.16 [0.52, 2.59]	0.726
Marital status		
Married	Reference	
Single	1.23 [1.05, 1.44]	0.011*

W, White; B, Black; AI, American Indian/Alaska Native; API, Asian or Pacific Islander; Low grade: grade I and II, high grade: grade III. *:P<0.05;**:P<0.01;***:P<0.001.

### Nomogram construction

**Figure 3:**
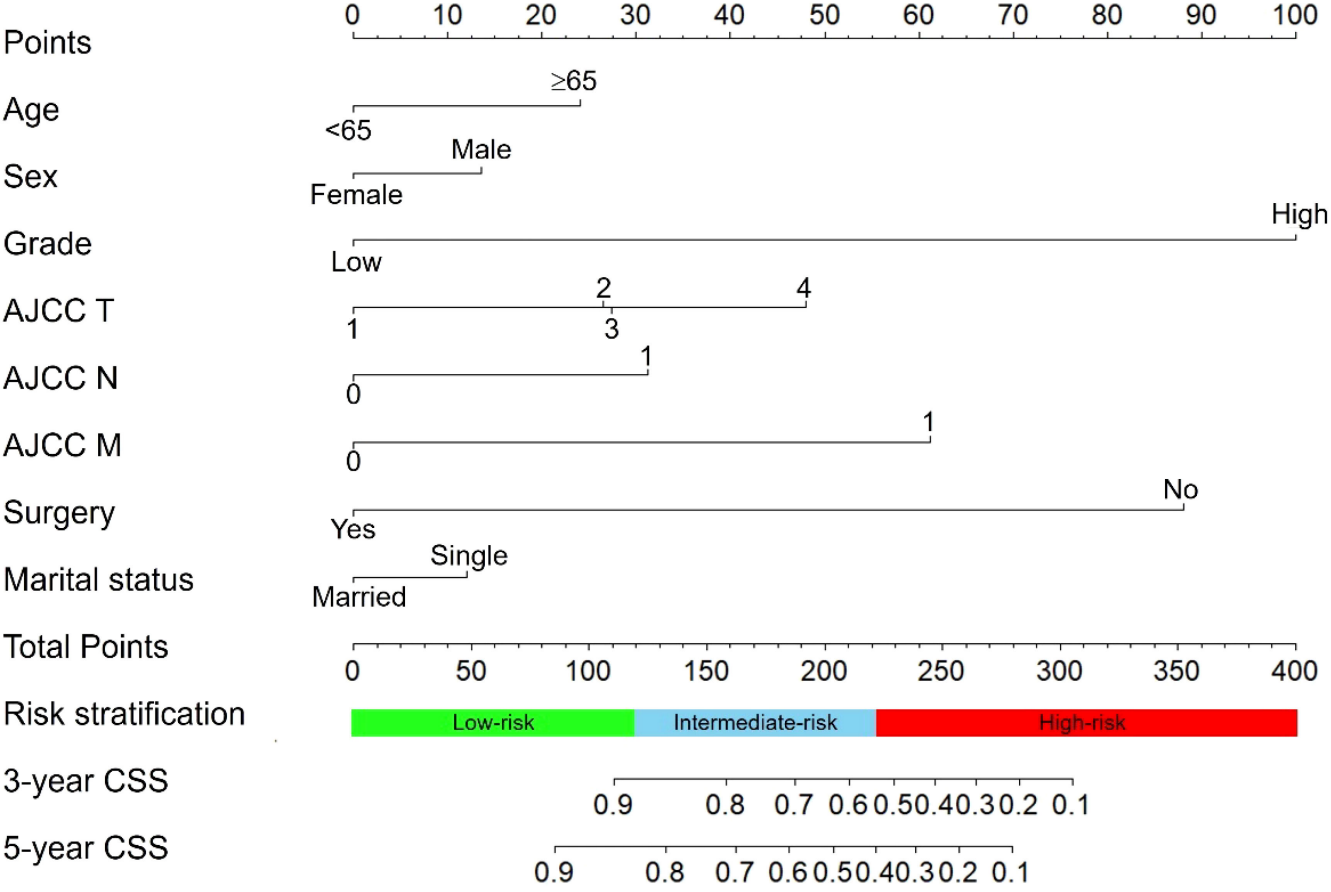
The nomogram predicting CSS in pNETs patients. Each factor was given a point on the basis of the nomogram. The total points were obtained by adding the given points of all factors. The estimated 3- and 5-year probabilities of CSS of the individual patient can be easily obtained from the nomogram based on the total points.

We constructed a nomogram for predicting the 3- and 5- years CSS of pNETs patients based on the independent prognostic factors from the training cohort (**Figure 3**). The nomogram indicated that tumor grade contributed the most to prognosis, followed by surgery, AJCC M, AJCC T, AJCC N, age, sex and marital status. When applying the nomogram for a single pNETs patient, the total score of the patient could be calculated by adding each score of the selected variables. Then the prediction of 3- and 5- years survival rates for the patient could be read on the nomogram.

### Risk stratification system

**Figure 4:**
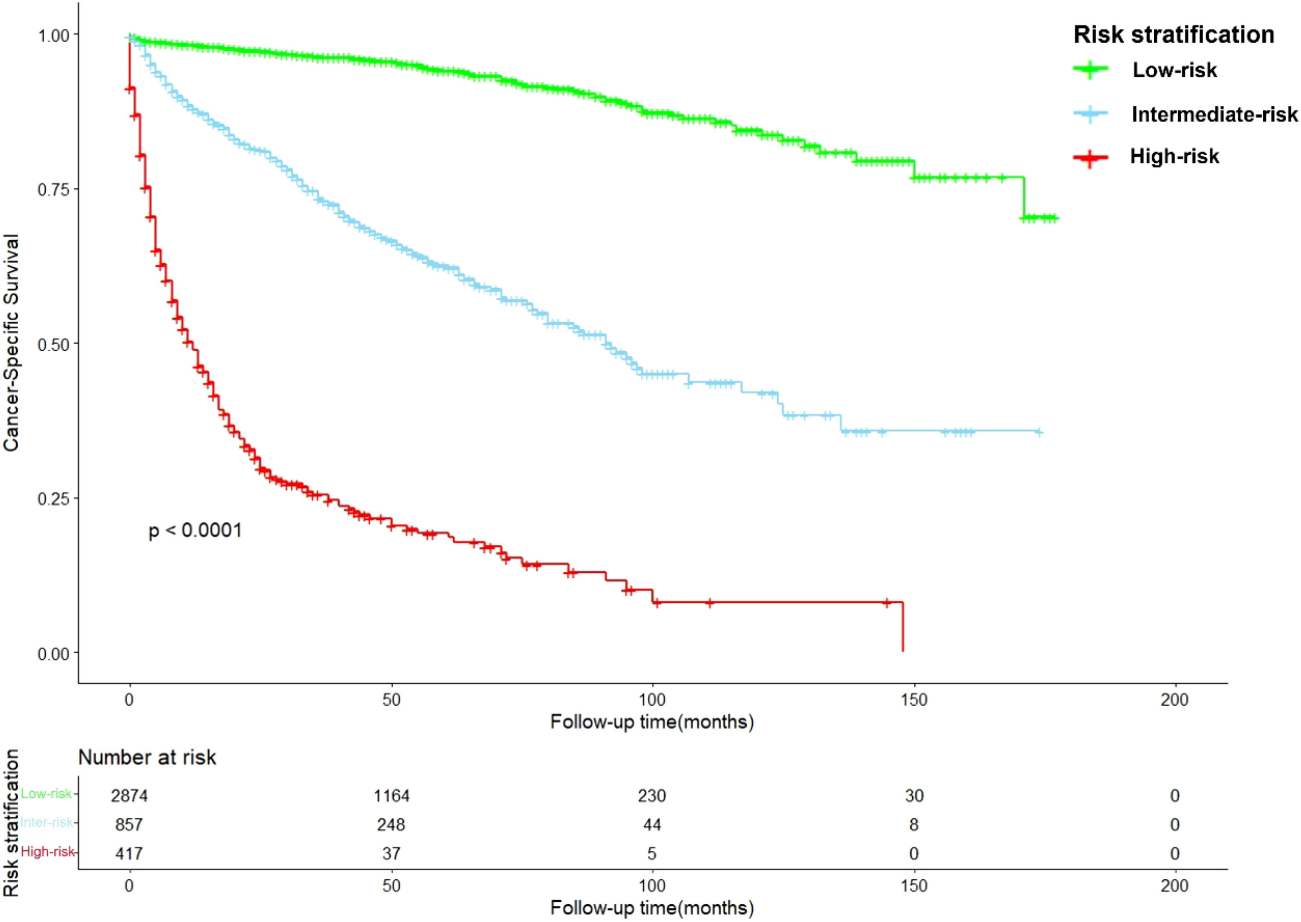
The Kaplan-Meier survival curves based on the risk stratification system.

After calculation by X-tile software (**Figure S1**), all patients were grouped into the low-risk (score: 0–121.2), intermediate-risk (score: 121.2–222.7), and high-risk groups (score: 222.7–378.1). Compared to low-risk group, HR of intermediate-risk group and high-risk group were 6.57 (P < 0.001) and 29.68 (P < 0.001). The C-index of the risk stratification system was 0.825. The Kaplan-Meier survival curves also showed the good discrimination power of the risk stratification system (**Figure 4**).

### Nomogram validation

**Figure 5:**
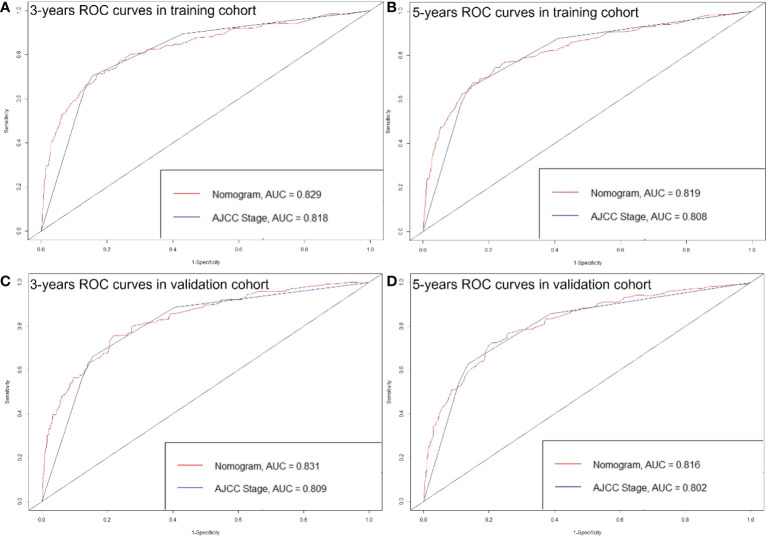
ROC curves of the Nomogram and AJCC stage in prediction of prognosis at 3- **(A)** and 5-year **(B)** point in the training cohort. ROC curves of the Nomogram and AJCC stage in prediction of prognosis at 3- **(C)** and 5-year **(D)** point in the validation cohort.

**Figure 6:**
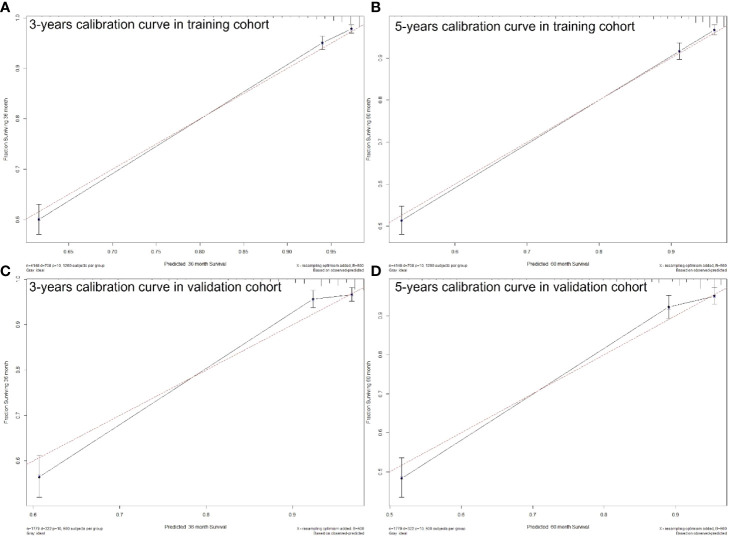
3- **(A)** and 5-years **(B)** calibration curves for probability of pNETs patients CSS nomogram construction in training cohort. 3- **(C)** and 5-years **(D)** calibration curves for probability of pNETs patients CSS nomogram construction in validation cohort. (Bootstrap = 500 repetitions).

The C-index of the nomogram was higher than which based on the AJCC staging system in both training cohort (0.856 vs. 0.781) and validation cohort (0.84 vs. 0.772). Also, the AUCs of the nomogram were higher than AJCC staging system in both training (3-year AUC: 0.829 vs. 0.818, 5-year AUC: 0.819 vs. 0.808, **Figures 5A, B**) and validation (3-year AUC: 0.831 vs. 0.809, 5-year AUC: 0.816 vs. 0.802, **Figures 5C, D**) cohorts for 3- and 5- years. The C-index and AUCs of the nomogram demonstrated that its superiority in discrimination over AJCC staging system. Furthermore, **Figure 6** showed the good consistency between predictions and actual observations when using the nomogram for pNETs patients.

## Discussion

Although the AJCC staging system is widely used for predicting prognosis in pNETs patients, it has inherent defects because it neglects many additional risk factors other than the TNM factors. Our study obtained 12 factors of pNETs patients from SEER database, and found 8 independent prognosis factors including: patient age, sex, race, tumor grade, AJCC T, AJCC N, AJCC M, surgery, radiation, chemotherapy, tumor function and marital status. Therefore, the C-index and AUCs of our nomogram were higher than which of AJCC staging system (C-index: 0.856 vs. 0.781; 3-year AUCs: 0.829 vs. 0.818 in training cohort).

A previous study demonstrated that younger than 60 years old and female were two protective factors for non-functional pNETs patients (16), though the results were not validated. In our study, over 65 years old (HR = 1.48, P < 0.001) and female (HR = 0.81, P < 0.01) were two independent prognosis factors for pNETs patients. Because 99.0% of pNETs in our study were non-functional, therefore the results in our study were consistent with previous one. The reason why female patients have better prognosis may be attributed to the protective effect of estrogen digestive tract tumors (17, 18).

The treatment for patients with pNETs was still controversial (19). Tumor resection was recommended for early-stage pNETs patients (20, 21), and our study verified that pNETs patients after surgery had a better prognosis than patients without surgery (HR = 0.26, P < 0.001). Radiation and chemotherapy could also be optional used for patients with unresectable pNETs, though the curative effect was not distinct enough (22). In our study, no significant protective effect was observed in pNETs patients who underwent radiation therapy (HR = 1.25, P = 0.07) or chemotherapy (HR = 1.15, P = 0.14).

Marital status has been found to have effects on the prognosis of many cancers such as bladder cancer (23) and renal cell carcinoma (24). It could be attributed to that single patients lack the emotional support and social interaction from partners and they are prone to have unhealthy lifestyles such as alcoholism, tobacco consumption and drug abuse. Our study also proved that single patients with pNETs had worse prognosis than patients in marriage (HR = 1.23, P < 0.05).

It was believed that patients with functional pNETs had a longer survival than those with nonfunctional pNETs (25–27). Due to the lack of specific symptoms, most of patients with nonfunctional pNETs could be diagnosed at a relatively advanced stage and had a worse prognosis. However, in our study, no significant prognosis difference was observed between functional pNETs patients and nonfunctional pNETs patients (P = 0.726). The reason could be the relatively small sample size of functional pNETs patients in our study (n = 62).

It should be noted that our study also has some limitations. First, this large-sample study was based on the SEER database, which may have some inherent biases. And our nomogram was internally validated, and it would be better to be validated externally using other populations.

## Conclusions

In conclusion, we constructed and validated a nomogram for predicting the 3- and 5-year CSS in pNETs patients. The proposed nomogram considered eight independent prognosis factors: patient age, sex, race, tumor grade, AJCC T, AJCC N, AJCC M, surgery, radiation, chemotherapy, tumor function and marital status. We have confirmed the precise calibration and excellent discrimination power of our nomogram. The predictive power of this nomogram may be improved by considering other potential important factors that we could not be obtained from the SEER database, and also by external validation.

## Data availability statement

The original contributions presented in the study are included in the article/**Supplementary Material**. Further inquiries can be directed to the corresponding author.

## Ethics statement

Ethical review and approval was not required for the study on human participants in accordance with the local legislation and institutional requirements. Written informed consent for participation was not required for this study in accordance with the national legislation and the institutional requirements.

## Author contributions

KL: conceptualization. GH and WS: data curation. YZ and BR: formal analysis. YL: supervision. KL: writing—original draft. All authors contributed to the article and approved the submitted version.
