# Supplementary material for: Construction and validation of a nomogram for patients with pancreatic neuroendocrine tumors: A population study of 5,927 patients
Source: Front Gastroenterol (Lausanne). 2023 Jan 10;1:1088133. doi: 10.3389/fgstr.2022.1088133 (PMC12952456; doi:10.3389/fgstr.2022.1088133)
Supplement: Supplementary file 1 [file DataSheet_1.docx]

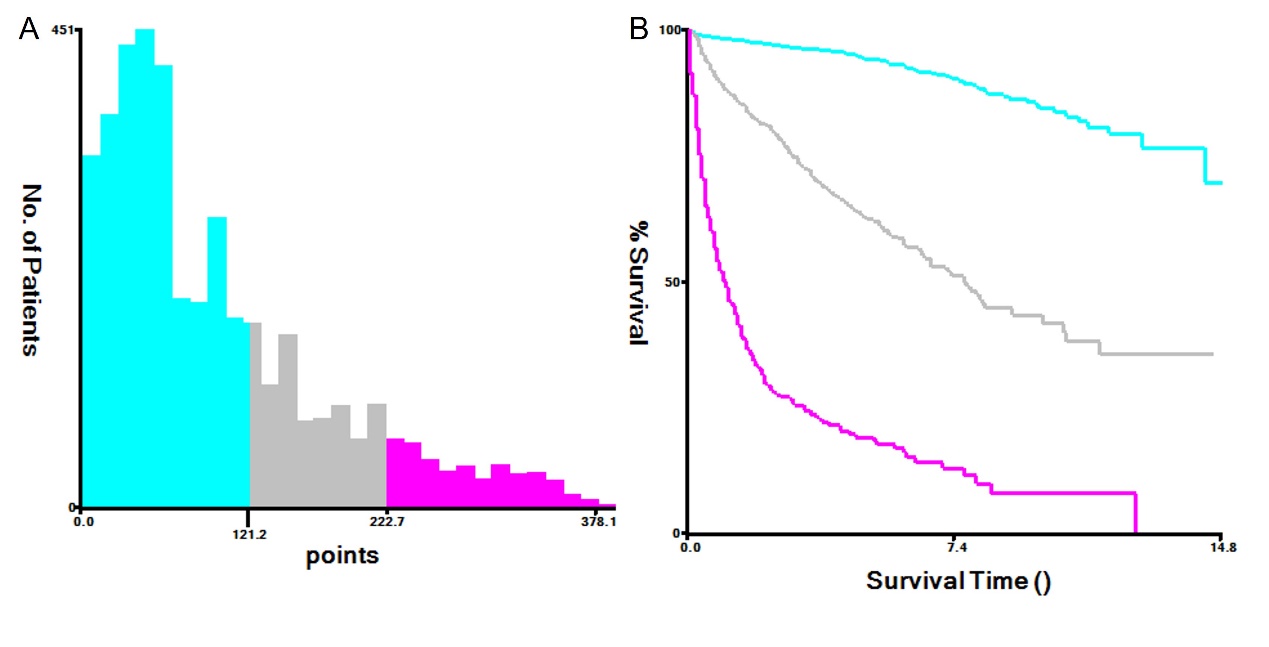


Figure S1. The cutoff value of the total points (A) and Kaplan-Meier survival curves (B) generated by X-tile software.
